# Effects of cervical transcutaneous Vagus Nerve Stimulation (ctVNS) on military cognitive performance during sleep deprivation

**DOI:** 10.3389/fphys.2025.1542791

**Published:** 2025-02-26

**Authors:** C. Bottenheft, Y. M. Fonken, L. F. Hendrikse, M. Koedijk, A. Landman, O. Binsch

**Affiliations:** ^1^ TNO Human Performance, Soesterberg, Netherlands; ^2^ TNO Learning and Workforce Development, Soesterberg, Netherlands

**Keywords:** vagus nerve stimulation, cervical transcutaneous vagus nerve stimulation, military cognitive performance, sleep deprivation, special operations forces

## Abstract

**Introduction:**

Maintaining cognitive performance during sleep deprivation is of vital importance in many professions, especially in high-risk professions like the military. It has long been known that sleep deprivation diminishes cognitive performance. To mitigate the negative effects on cognitive performance during crucial military tasks, new interventions are necessary. Non-invasive cervical transcutaneous vagus nerve stimulation (ctVNS) has gained traction as a method to boost alertness and cognitive functioning.

**Methods:**

We investigated the effects of a 2 × 2 minute ctVNS stimulation protocol on three cognitive tasks applied during conditions of sleep-deprivation: a psychomotor vigilance task (PVT), a multitasking task (SynWin), and an inhibitory control task (stop-signal task; SST). In addition, participants also performed a close-quarter-battle (CQB) test in virtual reality (VR) to examine if potential effects of ctVNS translate to operational military contexts. A total of 35 military operators from Special Operations Forces (SOF) and SOF support units participated. They were randomly assigned to an active stimulation group or sham group. Before stimulation at 19:00 h, participants performed baseline tests. Participants stayed awake through the night and performed the cognitive tasks every 3 h. The last round of cognitive tasks was followed by the VR test.

**Results:**

Though sleep deprivation was successfully induced, as evident from a decline in performance on all three cognitive tasks (effect of session: p < 0.001 SynWin; p < 0.001 PVT; p < 0.001 SST; Linear Mixed Model), no significant effects of ctVNS were found on cognitive task performance, as well as on the military operational VR task. However, the influence of stimulation intensity on SynWin performance showed a trend, indicating that higher stimulation intensities could have a negative impact on cognitive performance.

**Discussion:**

A 2 × 2 minute stimulation protocol may not be sufficient to elicit beneficial effects on cognitive-and operational military performance. Moreover, correct stimulation intensity may be critical to induce effects on cognitive performance, as stimulation effects may follow an inverted-u dose-response curve. Stimulation intensities in the current study are higher compared to a similar study that reported beneficial effects of ctVNS, which may explain this null finding. Further research is recommended to optimize stimulation protocols and investigate robustness of effects.

## 1 Introduction

Sleep deprivation is a critical issue that significantly impairs cognitive performance during execution of vital tasks in the military. Soldiers have to perform military tasks anytime and anywhere under sometimes severe circumstances, resulting in long duty hours and high-level sleep deprivation as a consequence. Sleep deprivation is known to negatively affect cognitive functioning, which is undesirable as soldiers often have to perform critical tasks while sleep deprived. Mitigating the effects of sleep deprivation is even more of importance when it involves soldiers of special operations forces (SOF) and SOF support units. Sleep deprivation is known to result in a general decrease of alertness and attention (Van Dongen and Dinges, 2005), such as brief moments of inattentiveness or drowsiness, slowed responses and wake-state instability (Alhola and Polo-Kantola, 2007). Numerous studies showed slowed reaction times on visual attention tasks, such as the psychomotor vigilance task (PVT; Hudson et al., 2020). Besides sustained attention or vigilance (Kusztor et al., 2019; Lim and Dinges, 2010), it is also known that sleep deprivation impairs prefrontal cortex-dependent functions (Chuah et al., 2006; Nilsson et al., 2005; Plieger and Reuter, 2020), including executive functioning (Aidman et al., 2019; Killgore et al., 2009; Kusztor et al., 2019; Nilsson et al., 2005). Executive functioning involve top-down processes that are responsible for goal-directed behavior (Hofmann et al., 2012) and can be divided in four main executive functions: inhibitory control, selective attention, working memory and task-switching (Friedman and Miyake, 2004; Diamond, 2013). Both maintaining vigilance and executive functioning are essential in high-risk professions and military contexts to detect threats and monitor critical systems. Besides this, executive functioning is crucial to suppress irrelevant or distracting information and to execute multiple tasks simultaneously. Overall, optimal cognitive functioning is necessary to enhance military readiness, safety, and mission success.

A promising new method to counteract this decrease in performance induced by sleep deprivation is to stimulate the nervous system with non-invasive Vagus Nerve Stimulation (nVNS) (Ridgewell et al., 2021; McIntire et al., 2021), which has been categorized as a Human Performance Augmentation intervention (Binsch et al., 2024). VNS involves applying a small electrical current to a branch of the vagus nerve. VNS has historically been applied invasively to treat depression and epilepsy. Patients with VNS implants have shown benefits to cognition in addition to therapeutic effects (Vonck et al., 2014), leading to an interest in non-invasive VNS in cognition research. A review by Ridgewell and colleagues showed that non-invasive auricular VNS seems to have a beneficial effect on cognition, particularly on complex executive functioning tasks (Ridgewell et al., 2021). In the military domain, non-invasive cervical (e.g., in the neck) VNS has been shown to improve vigilance and task-switching performance, as well as improving mood and wakefulness during sleep deprivation (McIntire et al., 2021). In this study, participants showed a smaller decrease in cognitive performance due to sleep deprivation when they received cervical transcutaneous VNS (ctVNS) stimulation at 7p.m. on day 1 of the study. This effect was most pronounced at 4–7a.m. following stimulation. The counter-fatigue effects shown in this study make ctVNS a promising intervention to support military cognitive performance under sleep deprived circumstances.

The underlying mechanism of how non-invasive VNS influences the brain is still largely unknown. VNS targets the vagus nerve, which influences brain regions involved in alertness, attention, and cognitive functioning by affecting the locus coeruleus (LC), the main source of norepinephrine (NE) (Vonck et al., 2014; Ross and Bockstaele, 2021). There is accumulating evidence that non-invasive VNS modulates LC-NE activity, as multiple studies have found biomarkers of activation of LC-NE activity, such as pupillary diameter and salivary alpha-amylase, as a result of (auricular) VNS (Giraudier et al., 2022; Mridha et al., 2021; Philips et al., 2024; Tona et al., 2020; Villani et al., 2022; Warren et al., 2017). Although the underlying mechanism of LC-NE system influences cognition while awake remains a matter of further investigation (Foote and Berridge, 2019), a limited number of promising studies suggest that non-invasive VNS has the potential to support military cognitive performance by promoting wakefulness, arousal, and cognitive functioning.

This study follows up on McIntire et al. (2021) to investigate the potential of non-invasive ctVNS to enhance cognitive and operational performance in SOF soldiers under conditions of sleep deprivation. This previous study showed a beneficial effect of ctVNS on multitasking performance as well as vigilance in a psychomotor vigilance task in an Air Force population (McIntire et al., 2021). Here, we study the effects of ctVNS on cognitive performance of SOF and SOF support units under conditions of sleep deprivation, including multitasking, vigilance, as well as response inhibition. Moreover, counteracting fatigue effects of ctVNS will also be tested in a virtual reality task deemed operationally relevant for SOF.

## 2 Materials and methods

### 2.1 Participants

A total of 35 healthy male soldiers from SOF and SOF support units were included in this study. They were recruited from two different military units experienced with sleep disruption during training, and all participants were familiar with close-quarters battle (CQB) procedures. *A priori* power analysis revealed that, with alpha (p) = 0.05, power (1-beta) = 0.8, two groups and five measurements, 24 participants should suffice to detect an effect of stimulation with an effect size of at least Cohen’s f = 0.23 (G*power software: Faul et al., 2007). One participant was excluded due to illness prior to receiving stimulation, leaving 34 participants, 10 more than the *a priori* power analysis calculated. Participants were randomly assigned (computer-based) to the sham (N = 15) or ctVNS group (N = 19). Group assignment was done beforehand, leading to imbalanced numbers between groups due to participant dropout. Approval for this study was granted by an accredited medical research ethics committee (MREC Brabant, reference number: P2316, approval number: NL84403.028.23). All participants gave written informed consent. Exclusion criteria were: atherosclerosis, other cardiovascular health issues, epilepsy and a history of psychiatric illness (including sleeping disorders) and having an active implantable (metallic) device. One week prior to starting the experiment, all participants were required to be, and remain, in the same time zone as the research center. The group of participants had an age range of 20–39 years old (M = 26.1, SD = 4.8). No serious adverse events were reported.

### 2.2 Study design

The study design was a single-blind, sham-controlled, intervention study, with Stimulation Group (Stim Group; ctVNS vs. sham) as between-subjects independent variable and Session (19:00h, 22:00h, 01:00h, 04:00h, 07:00 h) as within-subjects independent variable. Participants were randomly assigned to an active stimulation group, and a sham stimulation group. Participants and all researchers were blind to the type of treatment participants received, except for the researcher applying the stimulation (hence a single-blind study). The unblinded researcher was present for stimulation only, and was not involved in execution of the experiment otherwise. Participants were provided with information about the study at least 1 week in advance.

### 2.3 Procedure

**FIGURE 1:**
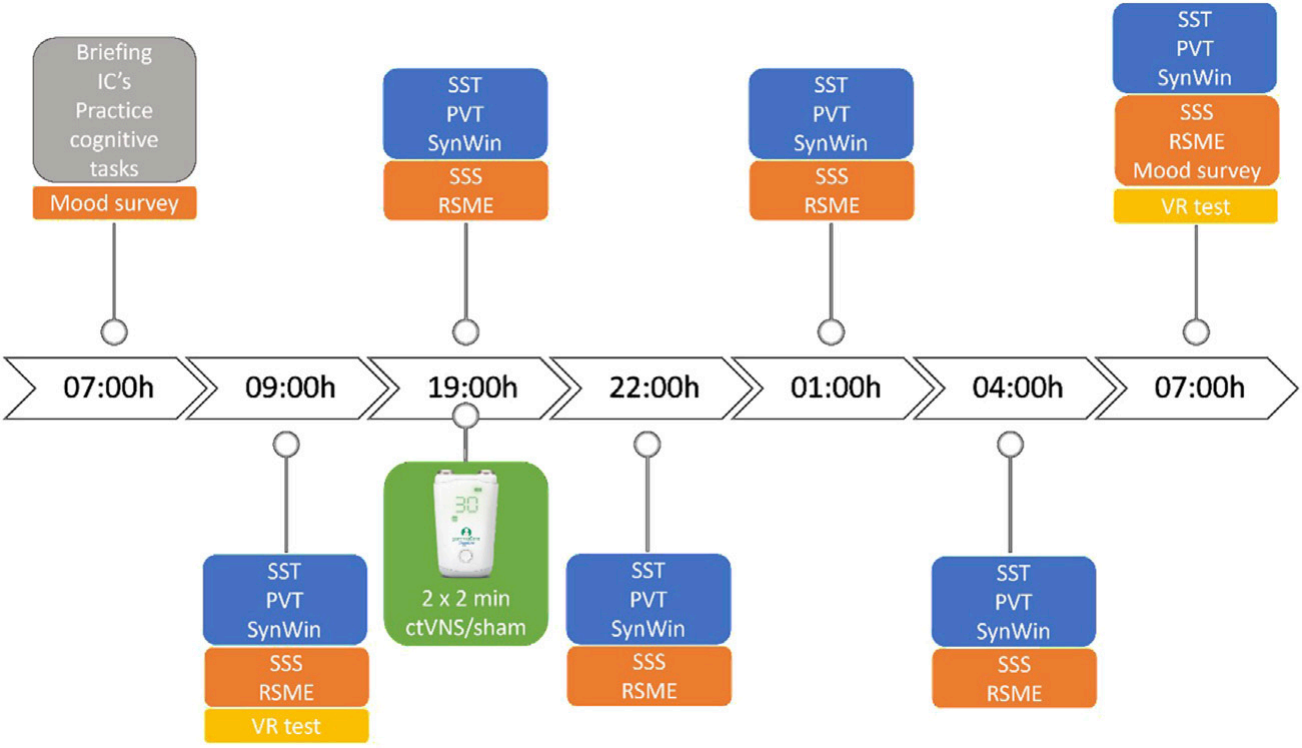
Timeline of study design. Neurostimulation was given 2 × 2 minutes at 19:00h, before the start of the cognitive tasks. IC = informed consent, VR test = virtual reality task, SSS = Stanford Sleepiness Scale, RSME = Rating of Subjective Mental Effort. Cognitive tasks consist of Stop-Signal Task (SST), Psychomotor Vigilance Task (PVT) and SynWin.

Military participants came in for approximately 24 h. Participants arrived at 07:00 h for a briefing, after which they signed the informed consent forms, practiced the cognitive tasks and received instructions about the Virtual Reality (VR) system. Soon after instructions, the participants performed the baseline tests, consisting of desktop cognitive tasks (Stop-Signal Task, Psychomotor Vigilance Task and SynWin), questionnaires and the VR test. They were expected back at 18:00 and in the meantime were instructed to spend their day normally without sleeping. Once back at the research site, the participants received ctVNS or active sham stimulation at 19:00 h. A medical doctor was present during stimulation procedures to assess (Serious) Adverse Events (AEs) and was available for consultation throughout the study. The cognitive tasks were performed immediately after the ctVNS or sham stimulation (at 19:00 h), and then after every 3 hours during the night until the next day (22:00h, 01:00h, 04:00h, 07:00 h). During the night of total sleep deprivation, participants were not allowed to take naps or consume caffeine or high-sugar-containing products. Standardized healthy snacks, low in sugar content were served during the night at the test location. At the last test (07:00 h morning day 2), participants performed the Virtual Reality test after they conducted the cognitive tasks (see Figure 1).

#### 2.3.1 ctVNS stimulator

Cervical transcutaneous vagus nerve stimulation (ctVNS) was applied with a CE-certified and FDA-approved gammaCore Sapphire™ device, originally developed for cluster headaches by electroCore™. The device applies a small electrical current transcutaneously through two electrodes. The researcher placed the device with conductive gel anterior to the sternocleidomastoid muscle in the neck of the participant, which overlies the cervical branch of the vagus nerve, and increased the electrical current gradually until a lip pull was visible. The neurostimulation device featured 40 intensity levels, which is assumed to linearly correspond to a maximum output of 60 mA/30V. For ctVNS, the minimum intensity used was level 20 (approx. 30 mA, assuming a linear output). For stimulation the gammaCore device produces five 5000 Hz sinusoidal pulses, repeated at 25 Hz. Stimulation was given on both sides of the neck for 2 minutes, separated by a 2-min pause. For sham stimulation, the gammaCore™ device was placed on the posterior neck (trapezius) muscle, the device was set to level 10 (approx. 15 mA), and was used without conductive gel.

#### 2.3.2 Cognitive tasks

Participants performed three cognitive tasks during each test session. The order of cognitive tasks was kept consistent to reduce variability in performance across participants unrelated to the intervention.

##### 2.3.2.1 SynWin

SynWin (originally called SynWork by Elsmore, 1994) was developed as a computer-based synthetic task to reflect the dynamic nature of a real-life work environment and measures the capability of multitasking. The operator is required to switch its attention between four tasks: memory task, arithmetic task, a visual monitoring task and an auditory monitoring task. These subtasks were presented in four quadrants (see Figure 1) on a laptop screen, and was performed for 20 min. For each subtask a score is calculated with points earned for correct responses, and points deducted for incorrect or missed responses. Points were deducted specifically for incorrect or missed identification in the memory task, incorrect calculation in the arithmetic task, allowing the fuel gauge to expire in the visual monitoring task, and auditory false alarms or misses. The sum of subtask scores was the multitasking total score, which was continually updated and presented to the participant in the middle of the screen. This score represents performance across all four subtasks by including points earned (+10) minus penalties for incorrect responses. The scores of the subtasks individually were stored but not presented to the participant.

##### 2.3.2.2 Stop-signal task

The stop-signal task is a form of Go/No-Go task that is designed to assess inhibitory processes (Logan et al., 1984). This desktop task consists of go-trials and stop trials. An arrow pointing left or right within a circle served as the go signal, instructing the participant to press the corresponding response keys (left/right). In 25% of the trials a stop-signal in the form of an auditory tone was presented after a short delay of between 50–1,150 m (stop-signal delay), instructing the participant to inhibit their response key-press. Stop-trials were titrated such that 50% of the time the person makes an error (pressing the response keys despite hearing the stop-signal) by flexibly increasing or decreasing the stop-signal delay by 50 m, depending on previous trial performance. The stop-signal delay was initiated at 250 m. The main outcome measure is the stop-signal reaction time (SSRT), which is calculated from the go-RT distribution and the average stop-signal delay. The SSRT is the amount of time that a person needs to successfully inhibit prepotent motor responses, and is a measure of inhibition and cognitive control capabilities (Logan et al., 1984). The task takes approximately 12 min to complete. The script for this task was downloaded from Millisecond (Millisecond Software, 2022).

##### 2.3.2.3 Psychomotor vigilance task

The psychomotor vigilance task (PVT) is designed to assess vigilance. This desktop task measures the speed by which participants respond to a visual stimulus (Thomann et al., 2014). Participants were instructed to press the response button (spacebar) as soon as the stimulus appeared on the screen. The stimulus was a red stopwatch counter in which the time was presented running in white numbers. The inter-stimulus interval, defined as the period between the last response and the appearance of the next stimulus, varied randomly from 2 to 10 s. The task had a duration of 10 min. A hit was defined as a correct response to the stimulus with a reaction time greater than or equal to 150 m and less than or equal to 500 m. Any response greater than 500 m was considered as a lapse and any response less than 150 m was classified as a False Alarm. Performance measures include accuracy (Hits/(Hits + False Alarms + Lapses)). This task takes approximately 12 min to complete. The script for this task was downloaded from Millisecond (Millisecond Software, 2022).

#### 2.3.3 Virtual reality test

The system used for the VR test was the Blacksuit system (RE-liON^©^, 2018, Enschede, the Netherlands). It has a wireless setup with full-body tracking and a head-mounted display (HMD). Participants were equipped with a replica of the Heckler and Koch HK416 and Glock 17. The HMD has a horizontal field of view (FoV) of 108° degrees and renders 60 frames per second (fps). The participants were able to physically walk around in a 30 × 30 m size space and see a virtual version of themselves and their weaponry. A military base in the Netherlands, was used for the experimental location to conduct the VR test. For a detailed description of the VR test, see Koedijk et al. (2024). During the VR test, 13 dichotomous items consisting of actions performed either correctly or incorrectly were obtained using a checklist constructed in advance with help of four Special Operations Forces (SOF) and three non-SOF CQB experts. The checklist was filled in by researchers after the testing finished, using recordings obtained via the After Action Review function of the VR system. The 13 items were combined into one performance measure consisting of the percentage of actions performed correctly.

#### 2.3.4 Subjective measures

Self-reported sleep information. Participants were asked about the number of hours they slept the night preceding the experiment and rated the quality of their sleep on a 5-point scale ranging from ‘Very bad’ (1) to ‘Very well’ (5).

Stanford Sleepiness Scale. The Stanford Sleepiness Scale (SSS; Hoddes et al., 1973) measures sleepiness as participants score how they feel on a 7-point scale ranging from ‘Wide awake’ (1) to ‘Falling asleep’ (7). Participants filled in the questionnaire before the baseline and during each session after baseline.

Mood questionnaire/POMS. The Dutch shortened Profile of Mood States (POMS; Wicherts and Vorst, 2004) consists of 32 mood-indicating adjectives with a Likert scale with five anchor points, ranging from 0 = ‘does not match your mood at all’ (0) to ‘matches your mood very well’ (4). The five scales of the shortened POMS are composed as follows: Tension (6 items; e.g., ‘nervous’), Depressed (8 items; e.g., ‘sad’), Angry (7 items; e.g., ‘grumpy’), 10 Vigorous (5 items; e.g., ‘full of energy’) and Tired (6 items; e.g., ‘exhausted’). Participants filled in this questionnaire upon arrival on morning 1 and before the last session at 07:00 h on morning 2.

Rating Scale for Mental Effort (RSME). Participants rated their perceived mental effort with The Rating Scale of Mental Effort (RSME) after each cognitive task (Zijlstra and van Doorn, 1985). The RSME scale ranges from 0 to 150, with higher values reflecting higher workload. It has nine descriptors along the axis, e.g., ‘not effortful’ at value 2, ‘rather effortful’ at value 58, and ‘extreme effort’ at value 112.

### 2.4 Data analysis

#### 2.4.1 Preprocessing

All posttest outcomes were transformed into delta scores by subtracting baseline performance from all other session performances. Participants whose performance deviated by more than two standard deviations from the grand mean in at least three sessions were excluded from the analysis. An additional exclusion criterium was applied to the SST data, where participants were excluded if they demonstrated race-model violations in at least three sessions. A race-model violation occurs when the mean unsuccessful stop reaction time is longer than the Go reaction time, which is an indication that the participant is not performing the task correctly. All analyses were conducted both with and without the outliers to determine whether the inclusion of outliers influenced the results and to ensure the robustness of the findings.

#### 2.4.2 Cognitive task performance, primary analysis

Group differences on baseline were analyzed using Wilcoxon rank-sum test. To test the effect of ctVNS on cognitive performance, Linear Mixed Models were used based on the nested within-subject design. Performance on all cognitive tasks were fitted in separate models, with the dependent variable being total score on the SynWin, SSRT on the SST, and accuracy on the PVT. Independent variables for all three models included Stim Group and Session. The interaction factor of Stim Group by Session was included as the fixed effect while subject ID was included as the random factor. Of primary interest were Stim Group main effect, indicating higher performance of one group throughout the study, and the interaction effect of Stim Group by Session, indicating higher performance of one group during one or more sessions. A Session main effect indicates a difference in performance due to the sleep deprivation manipulation. Data were analyzed in R (v 2023.12.1) using the *lme4* and *lmerTest* packages (Bates et al., 2015; Kuznetsova et al., 2017). F-statistics for linear mixed models were calculated using the *anova()* function within the *lmerTest* package and *p <* 0.05 was considered significant. If an interaction effect of Stim Group by Session was found, pairwise comparisons based on estimated marginal means were analyzed for each session to compare group performance using the *emmeans* package (Lenth, 2021).

#### 2.4.3 Cognitive task performance, secondary analysis

To test whether ctVNS had a differential effect on performance on the individual SynWin subtasks, another Linear Mixed Model was fitted including all delta subtask scores as dependent variables. The fixed and random factors remained the same as previously mentioned. Bonferroni correction for multiple comparisons was applied in the analysis of the subtasks scores.

#### 2.4.4 Cognitive task performance, exploratory analysis

In addition to the primary analyses focusing on the effect of ctVNS vs. sham, an exploratory analysis was conducted to investigate the role of stimulation intensity. There are inconsistencies in the application of stimulation parameters, such as frequency, intensity and timing, across studies (Olsen et al., 2023). This has resulted in a limited understanding of how these parameters influence outcomes and a standardized optimal protocol has yet to be developed. This exploratory analysis aimed to provide additional insights into how varying levels of stimulation intensity affect cognitive performance, potentially offering new perspectives on stimulation parameter protocols. Stimulation intensity level, ranged from 0 to 40, were documented for each participant for both the left and right side of the neck. If the stimulation intensity level varied between left and right, the mean of the two levels was calculated. Linear Mixed Models were fitted using performance on the cognitive tasks as the dependent variables and Stimulation intensity by Session as the fixed effect, including only participants who received active ctVNS. If participants were excluded in the primary analysis, they were similarly excluded in this exploratory analysis.

#### 2.4.5 VR test performance

To test for Stim Group differences, independent-samples t-test were performed using SPSS (Statistical Package for the Social Sciences) version 29.0 on the baseline-posttest delta scores of the percentage of actions performed correctly. To test for effects of sleep deprivation, paired-samples t tests were performed on the actions performed correctly during the baseline test and the posttest.

Subjective measures. To test for differences in sleep duration and sleep quality of the night preceding the experiment between Stim Groups, independent-samples-test were conducted in R (v 2023.12.1) using the built-in *stats* package.

To test Stim Group differences over time in sleepiness, delta SSS scores were analyzed with a 5 × 2 repeated measures ANOVA including factors: Session, and Stim Group. For the delta RSME ratings for the three cognitive tasks, a 5 × 2 repeated measures MANOVA was first performed with the factors: Session, and Stim Group. If Mauchly’s Test for Sphericity indicated a violation of the sphericity assumption for any of the main or interaction effects, the Greenhouse-Geisser correction was applied to adjust the degrees of freedom. This was followed-up by univariate repeated measures ANOVAs for each cognitive task if the MANOVA showed a significant main or interaction effect. These tests were conducted using the *rstatix* package (Kassambara, 2023). Significant main or interaction effects were followed-up by *post hoc* pairwise comparisons with Bonferroni correction. Lastly, independent-samples t-tests were performed on the delta POMS subscale scores to test for Stim Group differences in mood.

## 3 Results

The exclusion criteria resulted in the exclusion of two participants (one ctVNS) for the SynWin, two (one ctVNS) for the PVT, and four (two ctVNS) for the SST. One individual (VNS) was excluded from the analysis of all cognitive tasks while the other excluded participants were unique to each task. The number of participants in any group remained above 12 after outlier removal, which was sufficient to detect an effect according to *a priori* power analysis. The results of the analyses were consistent regardless of whether outliers were included or excluded, demonstrating the robustness of the findings. For total number of participants included for each individual task see Table 1. No (serious) Adverse Events (AEs) related to the study protocol were reported.

**TABLE 1:** Main and interaction effects of Group and Session on cognitive task outcomes using Linear Mixed Model models.

Task	N	Stim group	Session	Stim group x session
SynWin	32	(1.30) = 0.41, *p =* 0.53	(4,120) = 5.17, *p <* 0.001	(4,120) = 0.31, *p =* 0.87
Stop-Signal Task	30	(1.28) = 0.003, *p =* 0.96	(4,112) = 6.91, *p <* 0.001	(4,112) = 0.36, *p =* 0.84
PVT	32	(1.30) = 0.46, *p =* 0.50	(4,120) = 27.17, *p <* 0.001	(4,120) = 0.08, *p =* 0.99

### 3.1 Cognitive tasks

**FIGURE 2:**
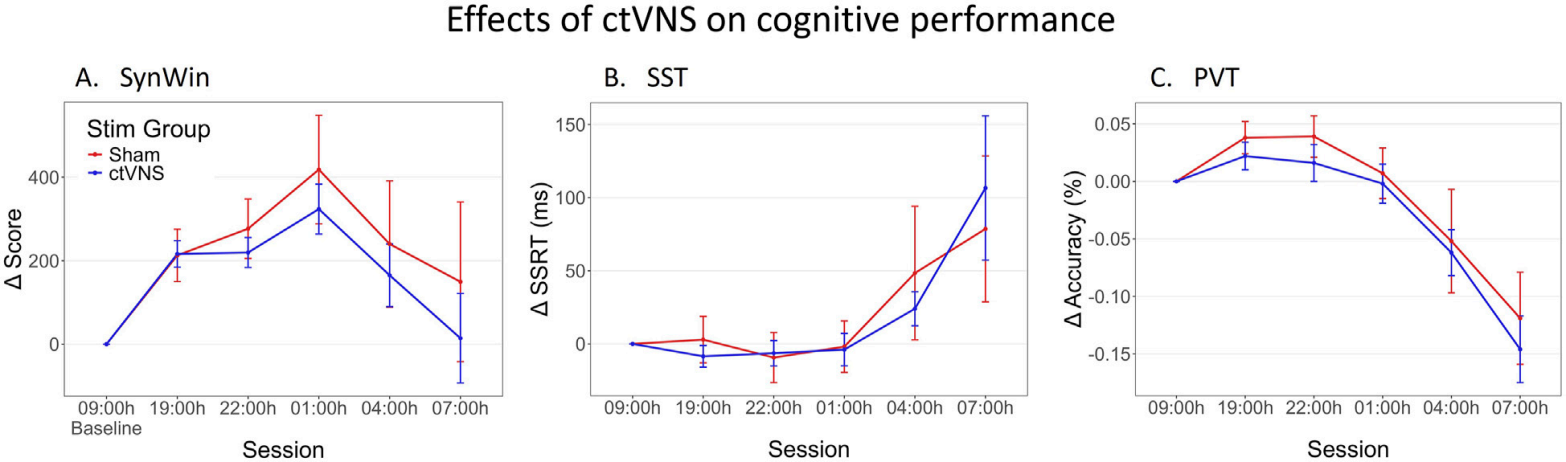
Change in performance on cognitive tasks during one night of sleep deprivation. Delta scores are calculated by subtracting the individual’s baseline score at 09:00 h from all other measurements, this means that measurements at 09:00 h are zero (datapoint included for visualization purposes). Mean delta score are calculated across participants, and are shown per stimulation group (red: Sham stimulation, blue: active ctVNS stimulation), across sessions (tasks were performed every 3 h between 19:00h and 07:00 h) on the SynWin **(A)**, Stop-Signal Task (SST) **(B)** and PVT (Psychomotor Vigilance Task) **(C)**. Error bars represent standard error.

Figure 2 shows task performance across sessions on the three different cognitive tasks. Performance on the SynWin task initially appears to increase, suggestive of a learning effect, before decreasing during the night. Performance on SST and PVT remain relatively stable for earlier sessions, before deteriorating during the night. Indeed, an effect of session was significant for all three tasks (see Table 1). However, no significant differences between active and sham stimulation groups are observed, nor are there significant interactions between stimulation group and session (Table 1). The Stim Groups did not appear to differ in task performance pre-treatment, as baseline performance did not differ between groups on any of the cognitive tasks (see Supplementary A for statistical results).

#### 3.1.1 SynWin subtasks performance

**FIGURE 3:**
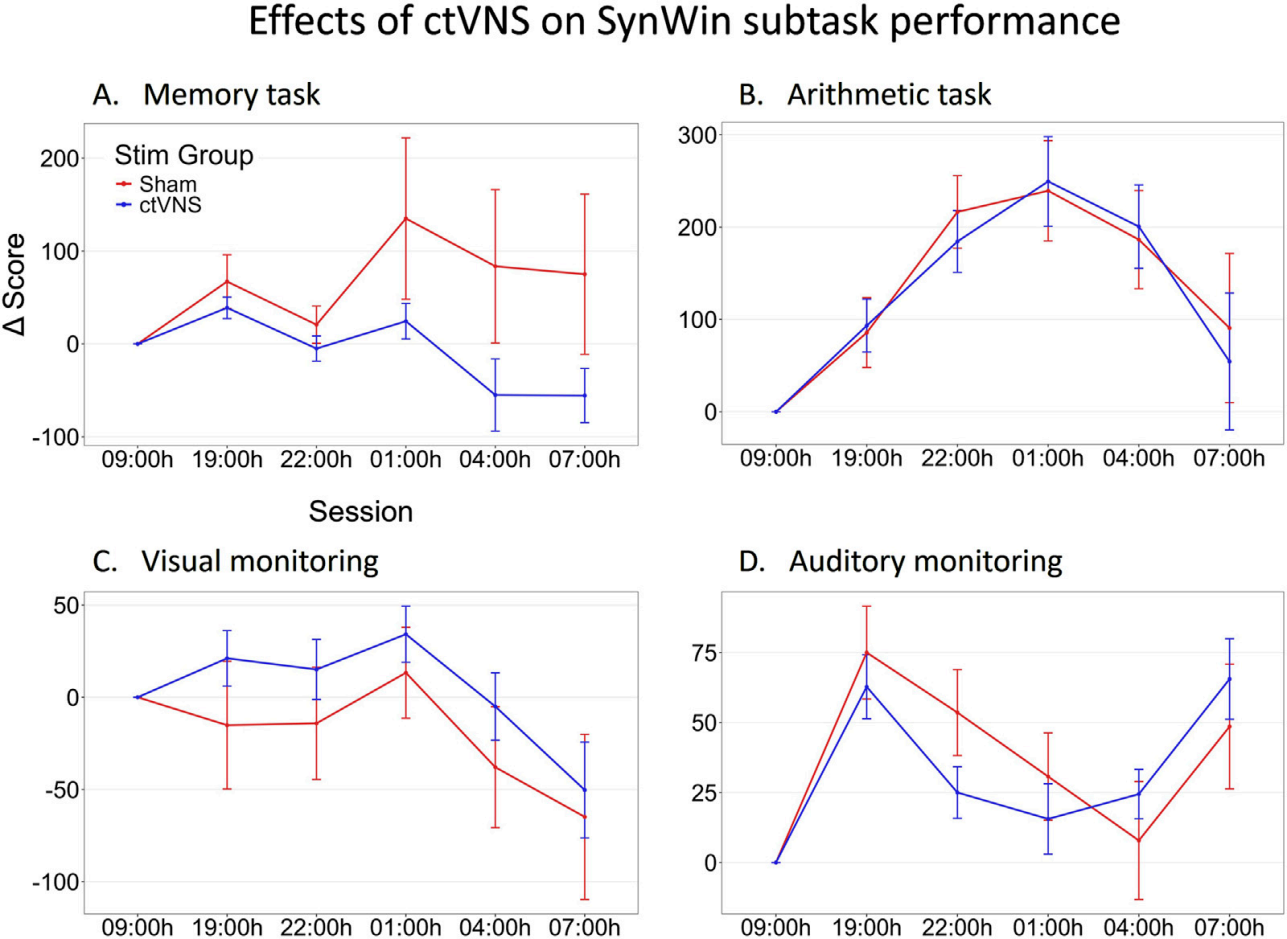
Change in performance on SynWin subtasks during one night of sleep deprivation. Delta scores are calculated by subtracting the individual’s baseline score at 09:00 h from all other measurements, this means that measurements at 09:00 h are zero (datapoint included for visualization purposes). Mean delta score are calculated across participants, and are shown per stimulation group (red: Sham stimulation, blue: active ctVNS stimulation), across sessions (tasks were performed every 3 h between 19:00h and 07:00 h) on the memory **(A)**, arithmetic **(B)**, visual monitoring **(C)** and auditory monitoring **(D)** subtasks of the SynWin. Error bars represent standard error.

The SynWin comprised of four subtasks that are performed simultaneously. These subtasks include memory, arithmetic, visual monitoring, and auditory monitoring tasks. Figure 3 illustrates task performance across sessions on the four subtasks of the SynWin. Performance on the arithmetic task increases, indicating a learning effect, before declining during the night. Memory task performance remains relatively stable in the earlier sessions, but varies between stimulation groups during the night. Performance on the visual monitoring task also stays stable for earlier sessions, then deteriorates during the night. For the auditory monitoring task, performance initially appears to increase due to a learning effect, decreases during the night, and then improves again the following morning. Indeed, the primary analysis involving individual Linear Mixed Model analyses revealed a significant main effect of Session on the arithmetic, visual monitoring, and auditory monitoring task, indicating a difference in performance on all SynWin subtasks related to the sleep deprivation manipulation. No main effects of Stim Group or interaction effects of Stim Group by Session (see Figure 3 for visual representation; Table 2 for statistical results) on any of the subtasks were found.

**TABLE 2:** Main- and interaction effects of Stim Group and Session on multitasking subtask performance using Linear Mixed Models. p-values are Bonferroni corrected for multiple testing.

Multitasking subtask	Stim group	Session	Stim group x session
Memory	(1.30) = 3.29, *p =* 0.32	(4,120) = 1.60, *p =* 0.71	(4,120) = 1.20, *p =* 1.25
Arithmetic	(1.30) = 0.01, *p =* 3.64	(4,120) = 12.60, *p <* 0.001	(4,120) = 0.34, *p =* 3.40
Visual monitoring	(1.30) = 0.76, *p =* 1.56	(4,120) = 7.54, *p <* 0.001	(4,120) = 0.16, *p =* 3.84
Auditory monitoring	(1.30) = 0.08, *p =* 3.14	(4,120) = 9.35, *p <* 0.001	(4,120) = 1.96, *p =* 0.42

#### 3.1.2 Exploratory analysis

The ctVNS device featured 40 intensity levels, the current study used a mean intensity level of 30.51 (SD = 4.48 arb. unit) for the ctVNS group while McIntire and colleagues used a mean intensity level of 20.55 (SD = 1.68 arb. unit) We thank L. McIntire (personal communication, October 2024) for sharing stimulation intensity data from McIntire et al. (2021).

**FIGURE 4:**
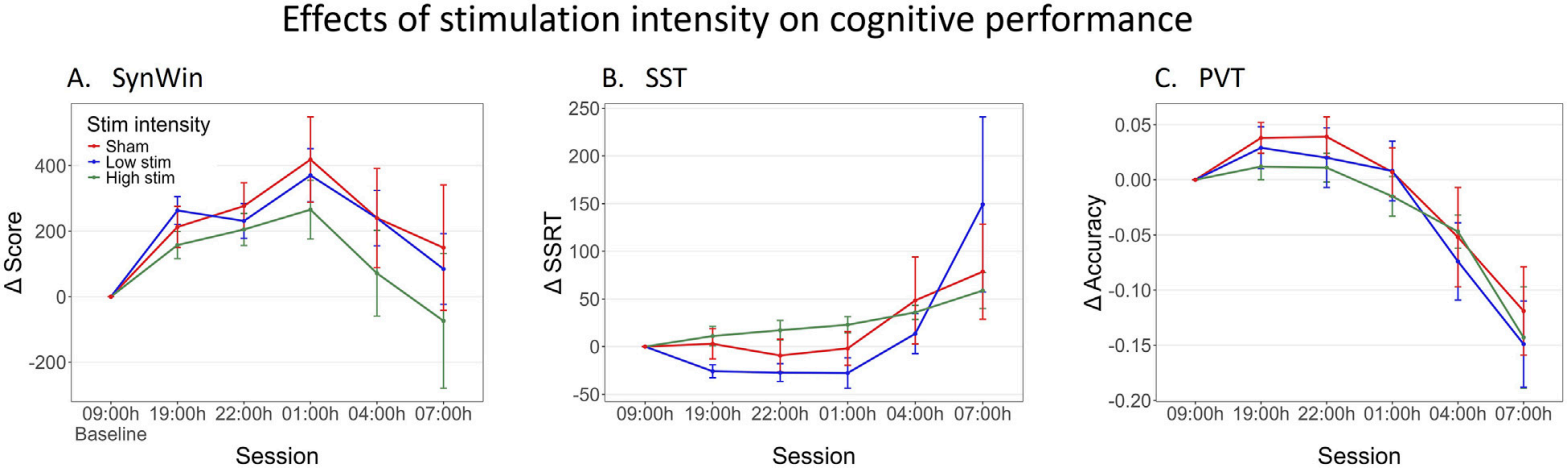
Change in performance on cognitive tasks during one night of sleep deprivation for sham, low-intensity stimulation and high-intensity stimulation Groups. For visualization purposes, the ctVNS groups were divided into two groups based on a median split. Delta scores are calculated by subtracting the individual’s baseline score at 09:00 h from all other measurements, this means that measurements at 09:00 h are zero (datapoint included for visualization purposes). Mean delta score are calculated across participants, and are shown per group (red: Sham stimulation, blue: low ctVNS stimulation, green: high ctVNS stimulation), across sessions (tasks were performed every 3 h between 19:00h and 07:00 h) on the SynWin **(A)**, Stop-Signal Task (SST) **(B)** and PVT (Psychomotor Vigilance Task) **(C)**. Error bars represent standard error.

For visualization purposes, the ctVNS group was divided into two subgroups based on a median split, again excluding the participant who was excluded on all the cognitive tasks in the primary analysis (Figure 4). The median intensity level was calculated to be level 30 (approximately 45 mA). Those receiving intensity level 30 or less were categorized into the *low stim* group (n = 10), while those who received a higher intensity level than 30 were placed in the *high stim* group (n = 8). The participants in the sham group remained unchanged (n = 15). However, the continuous values of Stimulation intensity of participants who received active ctVNS were used in this exploratory analysis. Figure 4 shows task performance across sessions on all cognitive tasks for different stimulation intensity groups, demonstrating similar shapes as in Figure 1. Linear Mixed Model analyses did not reveal a main effect of Stimulation intensity on the SST and PVT (see Supplementary B for statistical results). However, the analysis demonstrated a trend indicating that higher stimulation intensity levels were associated with lower performance on the SynWin (F (1,16) = 3.80, *p =* 0.07). Although this result did not reach statistical significance, it might indicate that higher stimulation intensities have a negative impact on cognitive performance.

### 3.2 VR test performance

VR test performance data was excluded for two participants (both in sham), who were instructed to stop due to their self-reported motion sickness symptoms reaching medium levels half-way into the baseline test. The average MISC score of the 32 remaining included participants was 1.28, SD = 2.02 in the pretest and 1.38, SD = 2.24 in the posttest. The delta VR performance (see also Koedijk et al. 2024) scores did not differ significantly between the groups, *t* (30) = 0.31, *p* = 0.761. The VR performance scores also did not differ significantly between the baseline test and the posttest, *t* (31) = 1.23, *p* = 0.229.

### 3.3 Subjective measures

Independent-samples t-test revealed no significant difference in self-reported sleep duration or sleep quality for the night preceding the experiment between the Stim Groups (duration: *t* (25.98) = −1.27, *p* = 0.21; quality: *t* (24.72) = −0.97, *p* = 0.34). For the sleepiness (SSS) ratings, the 5 × 2 repeated measures 5 × 2 ANOVA revealed a main effect of Session, F (2.96, 94.86) = 98.46, *p <* 0.001 (Greenhouse-Geisser corrected), a main effect of Stim Group, F (1, 32) = 4.23, *p =* 0.048, but no significant interaction effect, F (2.96, 94.86) = 1.38, *p =* 0.253 (Greenhouse-Geisser corrected) (see Supplementary Material C1 for a visual representation of the data). Unexpectedly, the effect of Stim Group demonstrated that the sham group rated their sleepiness significantly lower than the ctVNS group. Participants who received ctVNS showed a larger decrease in Vigour, *t* (31) = 2.21, *p* = 0.03, mean ctVNS = −8.26, mean sham = −5.43, on the mood (POMS) questionnaire. There were no significant differences before and after sleep deprivation between the Stim Groups for Tension, Depression, Anger or Fatigue on the POMS questionnaire (see Supplementary Material C2 for statistical results and visual representations of the data). A 5 × 2 MANOVA on the mental effort (RSME) ratings revealed a significant main effect of Session, *F* (12,21) = 2.59, *p* = 0.027, no significant main effect of Stim Group, *F* (3,30) = 0.17, *p* = 0.918, and no significant interaction effect, *F* (12,21) = 0.62, *p* = 0.806. Univariate ANOVAs on the effect of Session revealed a significant effect for the RSME scores during the Stop-signal task, F (2.89, 89.72) = 16.18, *p* < 0.001 (Greenhouse-Geisser corrected), the PVT, F (2.82, 87.52) = 12.85, *p* < 0.001 (Greenhouse-Geisser corrected), and the SynWin, F (2.60, 80.69) = 7.23, *p* < 0.001 (Greenhouse-Geisser corrected) (see Supplementary Material C3 for a visual representation of RSME scores per group).

## 4 Discussion

We performed a study on the potential of ctVNS as a measure to counteract a decrease in vigilance and cognitive performance under sleep deprivation circumstances. A group of military operators were kept awake one night while they performed standardized cognitive tasks every 3 hours, while receiving ctVNS or sham stimulation at 18 h the first day of the study. Cognitive tests assessed vigilance, multitasking, and response inhibition performance. In addition, participants performed close quarter battle scenarios in a Virtual Reality environment before and after sleep deprivation to assess effects of ctVNS on specific military operational performance. In our study we found no significant effects of ctVNS on cognitive performance in all three tasks, nor did we find effects in operational performance. This lack of an effect of ctVNS may be due to key parameters in our stimulation protocol. We did observe a decrease in performance on all tasks towards the end of the night, confirming that the sleep deprivation manipulation was successful.

### 4.1 No effects of ctVNS on cognitive performance

As mentioned, we did not find effects of ctVNS in any of the three cognitive tasks. This is contrary to findings of McIntire and colleagues, who reported beneficial effects of ctVNS on cognitive task performance (McIntire et al., 2021). This null result may be due to key differences in the stimulation protocols used in our study compared to the study by McIntire and colleagues (see sections ‘Stimulation protocol differences across studies’). The current study did not find stimulation effects on vigilance as measured by PVT accuracy, whereas McIntire et al. (2021) did observe that ctVNS improved PVT accuracy during sleep deprivation. Moreover, stimulation did not show a significant effect on the SynWin multitasking task, neither on the composite score, nor on any of the subtasks individually. McIntire et al. (2021) used the Multi-Attribute Task Battery (MATB, Santiago-Espada et al., 2011) to evaluate multitasking performance instead of the SynWin. Both tasks require participants to perform four different tasks simultaneously, but there may be subtle differences between both tasks. The effects of ctVNS on response inhibition under sleep deprivation, as measured with the Stop-Signal Task, have to our knowledge not been previously evaluated. The stop-signal reaction time (SSRT) proved sensitive to sleep deprivation. However, no effect of stimulation was found on SSRT. Finally, we did not observe effects of ctVNS on operational performance during a VR test. The VR test was included to assess if ctVNS effects could translate to operational performance measures. Since no effects were found on standardized cognitive tasks, which arguably should be more sensitive to interventions, it is to be expected that no effects are observed on the VR test either.

### 4.2 Stimulation protocol differences across studies: stimulation duration

The main difference in stimulation protocol in our study compared to the study by McIntire and colleagues is the total duration of stimulation. In our study, ctVNS was applied 2 × 2 minutes (4 min total) as specified by the manufacturer for treating cluster headaches. In contrast, McIntire et al. (2021) applied this 2 × 2 minute protocol twice in a row, totaling 8 min, with a 30-min interval between sessions. Our null findings suggest that perhaps a minimum duration of stimulation of >4 min total is required to produce measurable effects on cognitive performance. This expectation is supported since our stimulation protocol did not elicit effects on any of the tasks, including tasks previously shown to be sensitive to ctVNS manipulations (vigilance and multitasking).

### 4.3 Stimulation protocol differences across studies: stimulation intensity

Another key stimulation protocol difference between the two studies is stimulation intensity. The present study applied higher stimulation intensities compared to McIntire et al. (2021). Exploratory analysis investigating if stimulation intensity influences task performance showed no significant effects, although a trend was observed indicating that higher stimulation intensity levels were associated with lower performance on the SynWin. Previous studies have shown that VNS effects may follow an inverted-U relationship with stimulation intensity, as observed in animal literature (Buell et al., 2019), invasive VNS (as reviewed by Vonck et al., 2014), and non-invasive VNS (McHaney et al., 2023; Philips et al., submitted). These findings show that moderate levels of stimulation intensity can be more effective than higher stimulation intensities. For example, in epilepsy patients with invasive VNS, moderate levels of stimulation intensity have been shown to be most effective to enhance memory recall, whereas higher stimulation intensities seemed to decrease memory recall performance (Helmstaedter et al., 2001; Clark et al., 1999). For non-invasive (auricular) VNS, McHaney et al. (2023) showed that stimulation led to faster rates of learning, particularly with lower intensity stimulation (McHaney et al., 2023). For the purpose of cognitive performance augmentation in the military, we recommend further research into this inverted-U dose-response curve, to ensure optimal use of non-invasive VNS.

Another difference from the study by McIntire et al. (2021) is that in our study, ctVNS was administered by the experiment leader until a lip pull was observed. In contrast, participants in McIntire’s study self-administered the stimulation. When administered by the experiment leader, this could potentially result in higher intensities, as increasing the intensity continues until a visible lip pull is achieved. In contrast, participants self-administering the stimulation might have stopped increasing the intensity earlier, as they could feel the lip pull before it became visible. Moreover, it is unclear whether participants received the same intensity of stimulation, as it is unknown whether the device accounts for the impendence of tissue and skin. This variability in resistance could lead to differences in actual intensity experienced by each participant, potentially affecting the outcomes of the study.

### 4.4 An informative task battery for research into sleep deprivation

Although no effects of ctVNS were found in this study, it appeared that sleep deprivation was successfully induced in participants. In line with existing literature, the findings of the current study demonstrate that all three cognitive tasks were sensitive to the effects of sleep deprivation (Aidman et al., 2019; Lim and Dinges, 2010; Nilsson et al., 2005). Particularly at 04h and 07 h during the night, participants performed worse on key performance metrics in each task. For the PVT, sleep deprivation resulted in decreased accuracy, indicating a drop in vigilant attention and overall alertness. The SynWin multi-tasking test-battery, which requires simultaneous management of multiple tasks, also shows considerable performance decrements under sleep deprivation. Similarly, sleep deprivation negatively affected stop-signal reaction times, indicating reduced response inhibition and decreased ability to suppress prepotent responses. However, the virtual reality test did not show an effect of sleep deprivation (see Koedijk et al., 2024 submitted). This may be due to a learning effect canceling out any effects of sleep deprivation.

### 4.5 Limitations

The current study has several limitations. Firstly, we included military participants from two different units, each with varying levels of experience in sleep deprivation training and operational (VR) performance, which may have introduced variability in the results. In future research, a questionnaire should be used to control for the level of experience with sleep deprivation during training and operational VR performance. Another aspect that was not controlled for in this study was sleep vulnerability phenotypes (Hudson, Van Dongen, and Honn, 2020) and environmental factors. Additionally, some participants reported to the experimenter that they experienced low sleep quality for multiple nights prior to the experiment. Sleepiness ratings were significantly different between stimulation groups during the night, but were not different at baseline. However, it is possible that fatigue buildup leading up to the experiment only show up in sleepiness ratings during the night and not at baseline. For future studies we recommend to monitor, and control for, sleep quality for several nights prior to the experiment. Moreover, Smoking was not an exclusion criterium, and participants were allowed to use normal coffee intake during the day to preserve ecological validity. This could have influenced the results due to the cognitive-enhancing effects of nicotine and caffeine (Fiani et al., 2021; Levin, McClernon, and Rezvani, 2006). Finally, the sample included only male participants, as the military units from which they were recruited were predominantly male. It is key for further research to include women as well, as research in human and animal models has shown that ctVNS may affect women differently (Yaghouby et al., 2020; Yokota et al., 2022). These factors highlight the need for more controlled conditions in future research to ensure more reliable and valid results, for example, by using a within-subject design combined with strictly controlled environmental factors.

### 4.6 Conclusion

A 2 × 2 minute stimulation protocol may not be sufficient to elicit beneficial effects on cognitive- and operational military performance, and could potentially benefit from additional repetitions. Given the variability in stimulation protocols used across different studies, it is crucial for future research to systematically investigate the optimal parameters for eliciting robust effects. Specifically, future studies should focus on determining the appropriate duration, stimulation intensity, and number of repetitions required to achieve consistent and significant outcomes. By standardizing these variables, researchers can better understand the effects of stimulation and enhance the reproducibility of findings. Additionally, more research is needed to investigate the underlying mechanisms of ctVNS on cognition in healthy participants, which would provide more targeted and effective applications of this method. Such research will contribute to the development of more effective neurostimulation protocols. Furthermore, an inverted U-shaped stimulation response curve is proposed in literature to explain the effects of different stimulation intensity levels observed in various studies. This is relevant, because stimulation at high intensities may abolish beneficial effects of ctVNS, and may even be decremental. The findings of this study inform both the optimal use of ctVNS in military settings by counteracting fatigue, as well as contribute to the broader field of neurostimulation for cognitive enhancement. Furthermore, effective application of ctVNS has the potential to increase operational performance and military readiness. Finally, the authors would like to emphasize that VNS for cognitive enhancement should be seen mainly as a supplementary booster in challenging situations, rather than a replacement for adequate sleep, nutrition, and other essential preparatory activities.

## Data Availability

The raw data supporting the conclusions of this article will be made available by the authors, without undue reservation.
