# Supplementary material for: Effects of cervical transcutaneous Vagus Nerve Stimulation (ctVNS) on military cognitive performance during sleep deprivation
Source: Front Physiol. 2025 Feb 26;16:1542791. doi: 10.3389/fphys.2025.1542791 (PMC11897260; doi:10.3389/fphys.2025.1542791)
Supplement: Supplementary file 1 [file DataSheet1.docx]

# Supplementary

## Cognitive tasks results

Table 1. Wilcoxon sum-rank test results to test for Stim Group differences in baseline performance on the SynWin, Stop Signal Task (SST), and Psychomotor Vigilance Task (PVT).

| **Cognitive task** | **W** | **p-value** |
| --- | --- | --- |
| SynWin | 155.5 | 0.2705 |
| SST | 125 | 0.5579 |
| PVT | 93 | 0.2168 |

## Exploratory analysis results

Table 2. Main and interaction effects of Stimulation intensity and Session on cognitive task outcomes using Linear Mixed Model models. Only participants who received active ctVNS were included in this analysis.

| **Task** | **N** | **Stimulation intensity** | **Session** | **Stimulation intensity x Session** |
| --- | --- | --- | --- | --- |
| SynWin | 18 | (1,16) = 3.80, *p =* 0.07 | (4,64) = 0.41, *p* = 0.80 | (4,64) = 0.63, *p =* 0.64 |
| Stop Signal Task | 17 | (1,15) = 0.60, *p =* 0.45 | (4,60) = 0.51, *p* = 0.73 | (4,60) = 0.19, *p =* 0.94 |
| PVT | 18 | (1,16) = 0.03, *p =* 0.88 | (4,64) = 0.99, *p* = 0.42 | (4,64) = 0.96, *p* = 0.44 |

## Subjective measures results

The posttest data of the POMS were lost for a one participant in the ctVNS group. Three single data entries for the SSS (1.6% of total) and two data entries for the RSME (0.8% of total) were missing and imputed by substituting with the mean of the concerning treatment group in the concerning session. The participant who was excluded from the cognitive task analyses was additionally excluded from the RSME analysis.

### Sleepiness (SSS) ratings

An overview of the participants’ sleepiness ratings during all sessions is shown in Figure 1. In the remaining datasets, 5 participants had not given a rating during one session. The 5x2 repeated measures 5x2 ANOVA revealed a main effect of Session, F(2.96, 94.86) = 98.46, *p <* 0.001, a main effect of Stim Group, F(1, 32) = 4.23, *p =* 0.048, but no significant interaction effect, F(2.96, 94.86) = 1.38, *p =* 0.253.


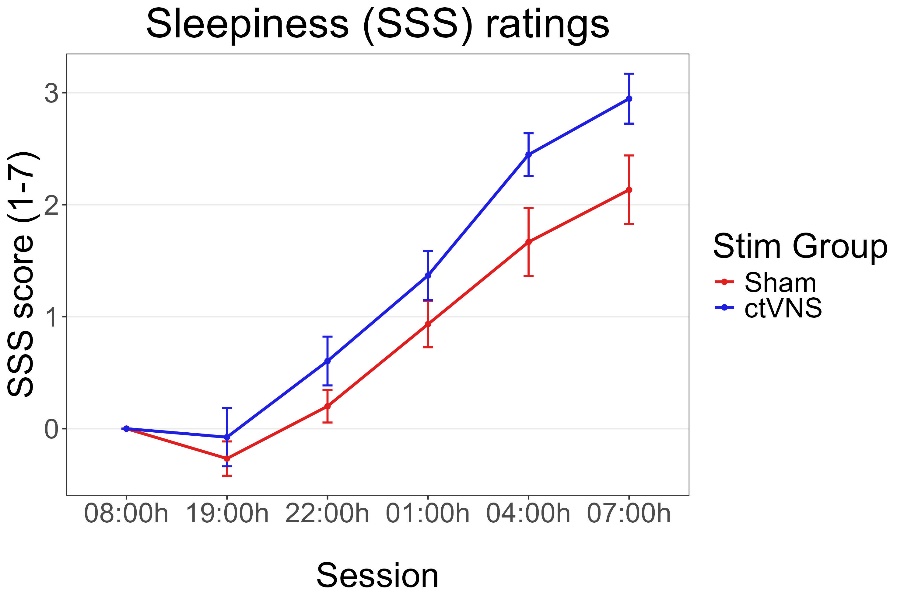


**Figure 1. Means and SEs of delta sleepiness (SSS) ratings of the Stim groups during one night of sleep deprivation.** Delta scores are calculated by subtracting the individual’s baseline score at 09:00h from all other measurements, this means that measurements at 09:00h are zero (datapoint included for visualization purposes). Mean delta scores are calculated across participants, and are shown per stimulation group (red: Sham stimulation, blue: active ctVNS stimulation), across sessions (SSS scores were rated every 3 hours between 19:00h and 07:00h) after performing cognitive tasks. Error bars represent standard error of the mean

### Mood (POMS) ratings

An overview of the mean scores on the five POMS subscales during the baseline session and final posttest session is shown in Figure 2. There was a significantly larger decrease in Vigour on the POMS for the ctVNS group than for sham, *t*(31) = 2.21, *p* = 0.03, mean ctVNS = -8.26, mean sham = -5.50. There was no significant difference between the groups for delta POMS Tension, *t*(31) = -0.58, *p* = 0.56, delta POMS Depression, *t*(31) = -0.45, *p* = 0.65, delta POMS Anger, *t*(31) = -0.07, *p* = 0.94, and delta POMS Fatigue, *t*(31) = -1.05, *p* = 0.30.


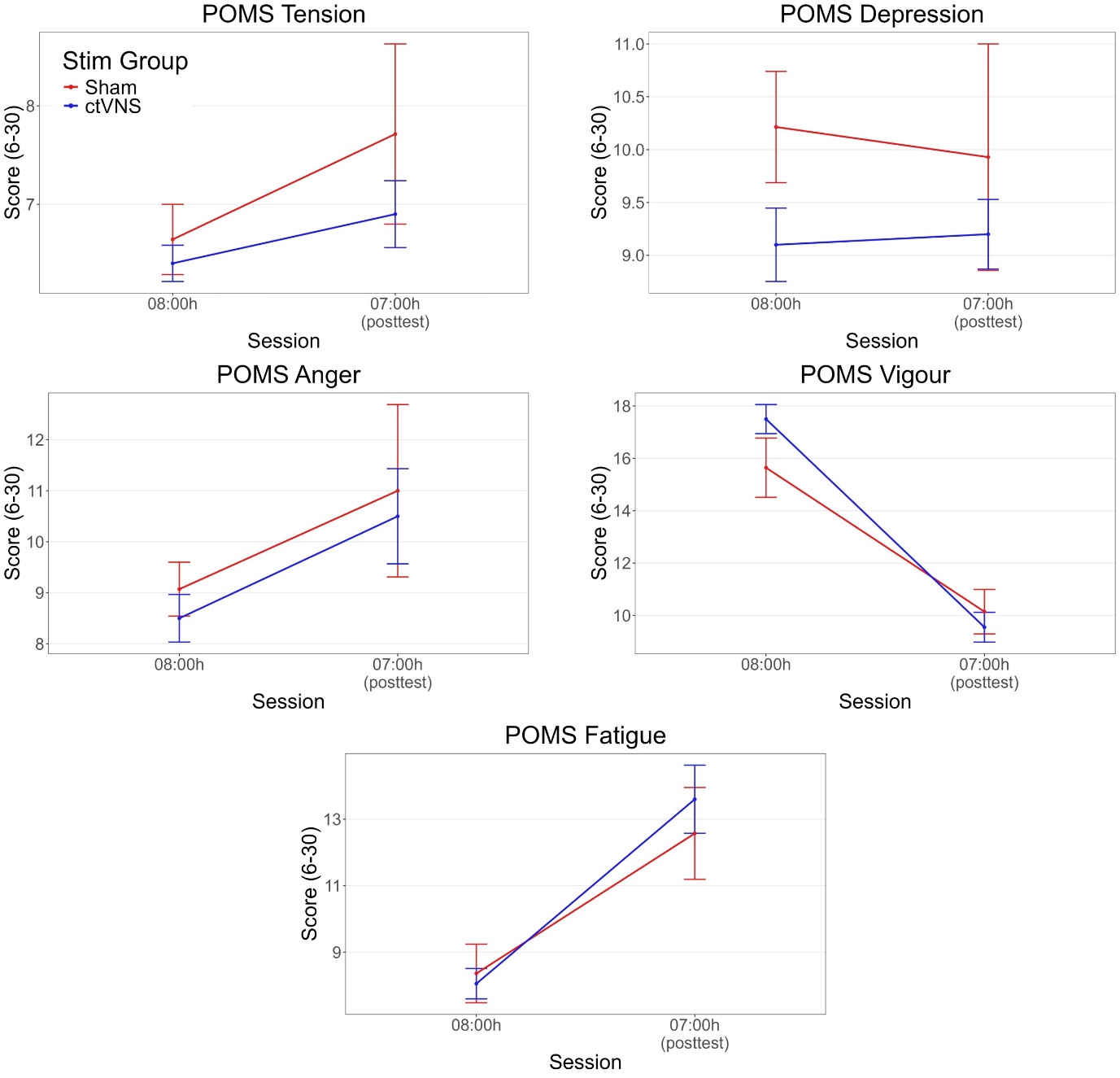


***Figure 2. Means of POMS subscales scores of the stimulation groups at baseline and final posttest sessions.*** *Error bars represent standard error bars. Note: values represented in this graph are absolute values instead of delta values.*

### Mental effort (RSME) ratings

**
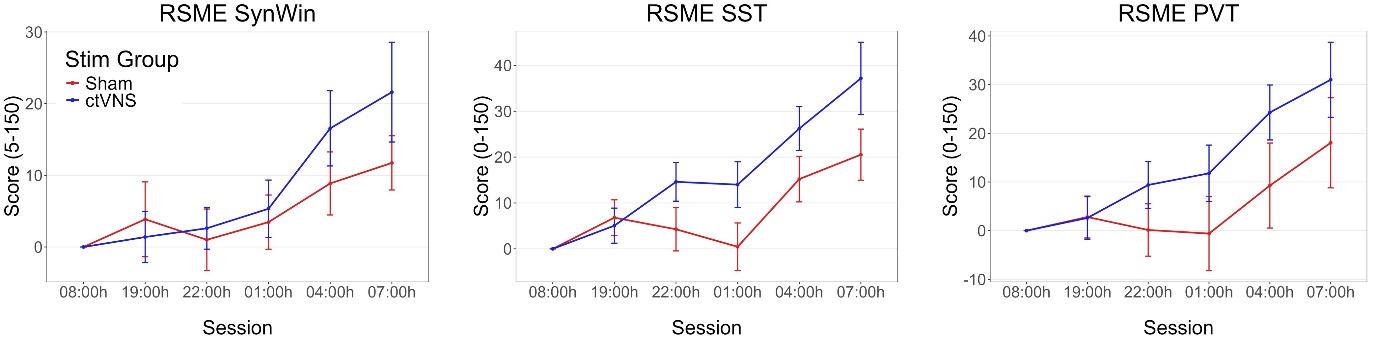
Figure 3.** **Change in RSME scores after performing cognitive tasks during one night of sleep deprivation.** Delta scores are calculated by subtracting the individual’s baseline score at 09:00h from all other measurements, this means that measurements at 09:00h are zero (datapoint included for visualization purposes). Mean delta score are calculated across participants, and are shown per stimulation group (red: Sham stimulation, blue: active ctVNS stimulation), across sessions (RSME scores were rated every 3 hours between 19:00h and 07:00h) after performing the SynWin, Stop Signal Task (SST), and PVT (Psychomotor Vigilance Task). Error bars represent standard error.
